# Personality Traits and Aggression as Explanatory Variables of Cyberbullying in Spanish Preadolescents

**DOI:** 10.3390/ijerph17165705

**Published:** 2020-08-07

**Authors:** Raquel Escortell, David Aparisi, María Carmen Martínez-Monteagudo, Beatriz Delgado

**Affiliations:** 1 Faculty of Education, International University of La Rioja, 26006 Logroño, Spain; raquel.escortell@unir.net; 2 Department of Developmental Psychology and Didactic, University of Alicante, 03080 Alicante, Spain; maricarmen.martinez@ua.es (M.C.M.-M.); beatriz.delgado@ua.es (B.D.)

**Keywords:** cyberbullying, personality, aggression, primary education

## Abstract

There is a growing interest in preventing cyberbullying in youth. However, multiple questions remain as to the relationship between cyberbullying and psychosocial variables. This study examines the relationship between personality traits, aggression and cyberbullying (victims, bullies, victimized bullies and not involved) in 548 Spanish students aged 10 to 13 (50.2% boys). To do so, the Screening of Peer Harassment, the Big Five Questionnaire for Children and the Aggression Questionnaire were used. Logistic regression analyses indicated that the extraversion trait is an explanatory factor for being a victim and openness is a protective factor against being a cyberbully. Agreeableness was found to be a positive predictor of being a cyberbullying victim. Only verbal aggression and anger were included as explanatory factors of being a victim and a victimized bully, respectively. The results are discussed, suggesting their potential implications in the development of preventive programs.

## 1. Introduction

Advances in Information and Communication Technologies have resulted in novel means of social interaction, creating a new world for young people that is ruled by electronic devices. The digital society in general and the immersion of youth in the same, have created numerous possibilities in only a few years’ time. However, it has also become a source of considerable danger, as is the case with cyberbullying [1]. Cyberbullying, also known as online bullying, has been defined as a form of harassment that involves the use of mobile telephones or the Internet to intentionally and repeatedly bully, threaten or intimidate others [2]. It manifests itself in a moderate prevalence of the roles of victims (30–40%), bullies (15–20%), victimized bullies (7–13%) [3,4] and with differences in terms of participation regarding gender (there are more female victims and more male bullies) [5] and age/school year, typically beginning during late Primary Education and peaking during the early years of secondary school [6,7].

The negative consequences experienced by youths involved in cyberbullying are extensive and may be quite debilitating, resulting, for example, in higher levels of school and social anxiety, lower self-esteem and academic goals, poorer academic performance and adjustment, school rejection behavior, substance use and suicidal ideation, etc. [4,8]. In addition, social interaction and personal and emotional adjustment problems, and conflictive and non-communicative family climate have been identified as predictors of cyberbullying during childhood and adolescence [9,10].

### 1.1. Personality and Cyberbullying Roles

Of the personal variables, personality traits have been analyzed with regard to cyberbullying roles. Descriptive studies have found that victims are characterized by higher scores on the traits of agreeableness (e.g., sensitivity towards others), openness to experience (e.g., various cultural interests, creativity and fantasy) [4,11,12,13], emotional instability or neuroticism (e.g., feelings of anxiety, fear, worry, low self-esteem and depression) and extraversion (e.g., sociability, enthusiasm, assertiveness and self-confidence) [4]. Bullies, on the other hand, have been characterized by lower levels of agreeableness and conscientiousness (e.g., order, precision and fulfilling of commitments), and higher levels of neuroticism [11,14]. So, personality traits play a major role as explanatory factors for behaviors of cyberbullying victimization and aggression. Therefore, predictive studies using regression analysis have found a significant relationship between cyberbullying and personality [4,11,12]. Celik, Atak and Erguzen [11] in a study with 230 young Turks that used the Ten-Item Personality Inventory [15], found that the greatest predictor of being a cyberbully was observing emotional instability in the victims, as well as presenting lower scores on the trait of openness to experience [11]. Festl and Quandt [12], using a sample of 408 German high school students and a reduced version of the Big Five Questionnaire [16], revealed that openness to experience predicted being a victim of cyberbullying. More recently, Rodríguez-Enríquez, Bennasar-Veny, Leiva, Garaigordobil and Yáñez [4], using the Big Five Questionnaire for Children [17] on a sample of 765 Spanish high school students, found that the probability of being a victim of cyberbullying increased as the levels of extraversion, neuroticism, openness to experience and agreeableness increased, while the conscientiousness personality trait was a protective factor against being bullied [4]. The not involved or bystander role consists of a heterogeneous group of individuals who are not directly involved in the cyberbullying events, but who may observe the behavior [18]. However, despite their importance in the perpetuation of the bullying episodes [19], their personality characteristics have yet to be analyzed.

### 1.2. Aggression and Cyberbullying Roles

Aggression, understood to be the combination of motor and behavioral (physical or verbal aggression), cognitive (hostility) and physiological-emotional (anger) components [20], is one of the personal characteristics that is the most closely related to scholar violence and cyberbullying, especially with respect to the role of the bully. Prior publications have suggested that bullies present high scores on general aggression [21,22]. These were also the findings in a sample of 3,349 Korean high school students, in which they found that the extended Internet use, past bullying episodes, high scores on aggression and low scores on self-control, served to predict the perpetration of cyberbullying [23]. On the other hand, using a sample of 849 German high school students, it was found that bullies or victimized bullies reported higher levels of behavioral aggression (proactive and reactive) than bystanders or students who were not involved in the bullying [18]. In addition, the probability of simultaneously being a bully and a victim increased as the adolescents demonstrated more aggressive behaviors and fewer social skills [18]. Therefore, aggressive adolescents tend to demonstrate their power and dominance over classmates through the use of force and intimidation, in this case, via technological resources.

As for the victims, they tend to demonstrate higher levels of anger [24,25], a component that is characterized by a state of excitation derived from conditions of threat or frustration, leading to uncomfortable emotions of varying intensities, from mild irritation to intense fury [26]. Other studies using samples of adolescents, have also found higher levels of aggression, anti-social behavior, anger and hostility in victims [27,28]. Giménez, Maquilón and Arnaiz [21], using a sample of 1,914 Spanish students, aged 11 to 21, found higher aggression levels in bullies and victims of cyberbullying when compared to those who were not involved in this behavior [21]. Aricak and Ozbay [29], using an extensive sample of 1257 Turkish high school students, reported that adolescents with high levels of anger had a higher probability of being bullies or victims of cyberbullying [29].

The role of the bystander, or the not-involved individual, has certain distinct behavioral profiles (in terms of aggression), given the heterogeneity of this group. So, it has found that “aggressive defenders” were more likely to develop reactive aggression (behavioral component), while “prosocial defenders” had a lower probability of developing reactive aggression [18]. Thus, there are apparently several distinct profiles within this not-involved individual role, based on the aggressive cyberbullying behaviors.

This evidence from past studies using adolescent samples, suggests that aggression increases the risk of victimization, with victims being more likely to demonstrate violent behavior (compared to non-victims). However, studies differentiating between victims and victimized bullies, affirm that the latter tend to be more aggressive when compared to the “pure” victims [30].

### 1.3. The Present Study

Given that cyberbullying is a relatively recent phenomenon, it remains necessary to clarify the psychosocial and emotional characteristics of the minors involved. Furthermore, although the use of the Internet is quite generalized in preadolescents (e.g., more than 90% of Spanish children, aged 10 to 13, have access to the Internet [31], and its incidence has been demonstrated over the last school years of Primary Education [6], most of the prior publications have focused on secondary school students, with few studies using samples from primary school and with limited data on the role of the not-involved individual or bystander. Therefore, this study proposes the analysis of a sample of Spanish preadolescents (aged 10 to 13), considering differences in scores on personality and aggression in victims, bullies, victimized bullies and not-involved individuals, as well as the explanatory power of both variables in predicting participation in each of the cyberbullying roles.

Based on the empirical evidence mentioned above, it is expected to find that victims will have higher scores on the traits of extraversion, cordiality and openness to experience, and it is anticipated that openness to experience will serve as a significant predictor of being a victim (Hypothesis 1). As for aggression, it is anticipated that victims will have higher levels of anger and hostility as compared to those who are not involved (Hypothesis 2). In terms of the roles of bullies and victimized bullies, it is expected that these individuals will have lower scores on openness to experience, agreeableness and conscientiousness, and that their low levels of openness to experience would explain their participation in cyberbullying (Hypothesis 3). Furthermore, it is anticipated that bullies and victimized bullies, would have higher scores on aggression and that these scores would explain the cyberbullying (Hypothesis 4). Finally, with respect to the role of the not-involved individuals, an open hypothesis has been proposed, given the lack of evidence on the relationship with personality (Hypothesis 5), while it is expected that their scores on aggression will be significantly lower than those of the other roles (Hypothesis 6).

## 2. Materials and Methods

### 2.1. Participants

The reference population consisted of 5th and 6th grade students from the Spanish province of Alicante (Spain). Of the 108,002 students enrolled in the primary schools in the province, four public schools and two private schools were randomly selected, with the recruited sample consisting of 558 students, of which six were eliminated due to response errors or omissions and four were excluded due to the failure to provide a parental informed consent for study participation. So, the total sample consisted of 548 students (50.2% males) aged 10 to 13 (*M* = 10.95; *SD* = 0.75): 276 (50.4%) from 5th grade and 272 (49.6%) from 6th grade of primary school.

The *χ*^2^ test was used to analyze the homogeneity of the sample, considering the gender and school year, finding no statistically significant differences between the four groups of Gender x School Year (*χ*^2^ = 2.50; *p* = 11).

### 2.2. Instruments

#### 2.2.1. Screening of Harassment Among Peers (SPH)

This is a self-reporting tool [32] that assesses 15 harassment behaviors, carried out via electronic means (e.g., by sending offensive or insulting messages, making offensive calls, spreading photos or videos over YouTube, making anonymous calls to frighten, threaten or blackmail). Although the questionnaire also measures bullying behavior, for this study, only the subscale measuring cyberbullying was used, consisting of 45 questions (“Have they sent offensive or insulting messages to you via mobile or internet?”) that are answered using a 4-point, Likert-like scale ranging from 0 (never) to 3 (always). A triangular response system is used, since the assessed individual should identify whether or not he/she had ever suffered from bullying behaviors as a victim, if he/she has engaged in these behaviors as a bully or if he/she has observed them being carried out by others during the past year.

The test offers 4 scores on cyberbullying: victimization (victimization behavior suffered), aggression (bullying behavior carried out on others), observation (bullying behavior that has been observed by the assessed individual, carried out on others) and aggression–victimization (includes the level of victimization and the level of perpetration, that is, bullying behaviors suffered as a victim and carried out as a bully). Based on these scores, the questionnaire permits the identification of the roles of victims, bullies, victimized bullies and bystanders. The psychometric studies performed by the original authors confirmed the test’s suitable internal consistency (α = 0.91) and a structure consisting of three factors that explain 40.15% of the variance [33]. Similarly, other publications have supported the instrument’s reliability and validity [3,34]. The internal consistency of the subscales used in this study were found to be adequate: Victimization (α = 0.94), Aggression (α = 0.96), Aggression-Victimization (α = 0.98), and Observation (α = 0.95).

#### 2.2.2. The Big Five Questionnaire for Children and Adolescents (BFQ-C)

An instrument [17,35] consisting of 65 items designed to measure personality in children and adolescents, based on the Big Five model [36] and designed around the five dimensions: Extraversion (“I want to see others”), Agreeableness (“I am decent and honest with others”), Conscientiousness (“I place a lot of effort into what I do”), Neuroticism (“I am sad”) and Openness to experience (“I understand things quickly”). The questionnaire items are responded to using a Likert-like scale having five alternative responses (1 = almost always; 5 = almost never). The BFQ-C was created by [17] and was adapted to the Spanish population by [35] using a sample of 852 children aged 8 to 15, demonstrating adequate levels of validity and internal consistency (α = 0.78–0.88). Adequate rates of internal consistency (Cronbach’s alpha) have been found for the BFQ-C subscales in this study: 81 (Extraversion), 86 (Neuroticism), 86 (Openness), 91 (Conscientiousness), and 95 (Agreeableness).

#### 2.2.3. Aggression Questionnaire (AQ)

This instrument [20,37] consists of 29 items referring to aggressive behaviors and feelings, coded with a 5-point Likert-like scale (1 = completely false for me; 2 = completely true for me). The questionnaire consists of four scales: Verbal aggression (“my friends say that I argue a lot”), Physical aggression (“I tend to get into fights”), Hostility (“sometimes I get very jealous”) and Anger (“I get so angry that I feel like I could explode”), which assess the three components of aggression: motor/behavioral (physical and verbal aggression), cognitive (hostility) and physiological–emotional (anger). The AQ was developed based on the Hostility Inventory [38] and a Spanish adaptation was created by [37] using a sample of 1382 high school students. The results indicated a structure consisting of four factors, through factorial analysis that explained 46.37% of the overall variance, with a satisfactory reliability of the scores (α ≥ 0.86). Acceptable internal consistency coefficients were found for the study’s AQ scores: Physical aggression (α = 0.77), Verbal aggression (α = 0.74), Anger (α = 0.64), Hostility (α = 0.75) and overall score AQ (α = 0.90).

### 2.3. Procedure

Following an interview with the school management team and having requested the relevant permits from the educational authorities, the study objectives were described, and the signed authorization was requested from the parents of the minors. Questionnaires were completed on a voluntary basis, collectively, during a class period. The anonymity of the participants was guaranteed by using identification numbers on the response sheets. Researchers were present throughout the testing period in order to clarify any potential doubts and to ensure proper test administration. Average administration times were 15 min (SPH), 20 min (BFQ-C) and 10 min (AQ). Standards regarding research on humans were respected, in accordance with the ethical principles of the Declaration of Helsinki and the school’s Ethics Committee (UA-2018-02-21).

### 2.4. Statistical Analyses

After grouping the sample into victims, bullies, victimized bullies and not involved (Screening of Peer Harassment) [32], an analysis of variance (ANOVA) study was carried out as well as post hoc Bonferroni test, to analyze differences in personality and aggression between the distinct cyberbullying roles. Then, the effect size was calculated using Cohen’s *d* [39]. Interpretation of the effect size is quite simple: values lower than or equal to 0.20 indicate a very small or insignificant effect size, those between 0.20 and 0.49 are considered to be small, those between 0.50 and 0.79 are moderate and those exceeding 0.80 are considered to be large [39]. Finally, in order to assess the potential predictive relationship of personality and aggression with cyberbullying, a forward stepwise logistic regression analysis was carried out, based on Wald’s method. To estimate the goodness of fit of each model, the percentage of correctly matched cases was determined, as well as Nagelkerke’s R^2^. Quantification of the probability of the appearance of an event (e.g., being a cybervictim) was carried out using the Odds Ratio (*OR*). SPSS 23.0 (IBM Corporation) was used for ANOVA and logistic regression analysis.

## 3. Results

### 3.1. Differences in Personality and Aggression in Victims, Bullies, Victimized Bullies and Not-Involved Individuals in Cyberbullying

Tests of differences in means indicate that bullies and those who are not involved in the cyberbullying had significantly lower scores on extraversion, agreeableness, conscientiousness and openness to experience and significantly higher scores on neuroticism (see Table 1). The effect size was small for the extraversion factor between the role of the not-involved individuals and the roles of victims (*d* = 0.38) and victimized bullies (*d* = 0.32). As for agreeableness, the effect size of the differences found was moderate (*d* = 0.53) between the roles of bully and victimized bully, and was small (*d* = 0.38) between the roles of victimized bully and not involved individuals. For the conscientiousness factor, the effect size was small (*d* = 0.32) between the roles of victimized bully and not involved. For neuroticism, the effect size was small between the role of not involved and bully (*d* = 0.10) and victimized bully (*d* = 0.38). In the case of the openness factor, the effect size was small between the role of the not involved individual and the roles of victim (*d* = 0.36) and victimized bully (*d* = 0.40), with a moderate effect size in the difference between the role of bully and the roles of victim (*d* = 0.57) and victimized bully (*d* = 0.55).

As for aggression, students who were not involved in the cyberbullying revealed significantly higher scores on the scales of verbal aggression and anger. In the case of verbal aggression, the effect size was moderate (*d* = 0.49) between the roles of victim and not-involved individual, and also moderate (*d* = 0.48) between the roles of victimized bully and not-involved individual. As for anger, the effect size was moderate (*d* = 0.66) between the roles of victimized bully and not-involved individual. For the scores on hostility and physical aggression, no statistically significant differences were found between the distinct cyberbullying roles.

### 3.2. Prediction of Being a Cyberbullying Victim

Based on logistic regression analysis, it was possible to create two predictive models for being a cyberbullying victim based on personality and aggression (Table 2), with 77.8% (χ^2^ = 6.02; *p* = 0.00) and 89.4% (*χ*^2^ = 6.64; *p* = 0.00) of the cases being correctly classified, respectively. The goodness of fit (Nagelkerke’s R^2^) for both models was 0.03 and 0.02, respectively. The OR indicates that students are 3% more likely to be a victim of cyberbullying (as compared to the not involved group) with every one-unit increase in their score on the extraversion trait and are 11% less likely to be victimized with every one-unit increase in their score on verbal aggression.

### 3.3. Prediction of Being a Cyberbully

A model was obtained to predict being a cyberbully based on personality (see Table 3), permitting the estimation of 93% of the cases (*χ*^2^ = 4.12; *p* = 0.00), with a goodness of fit (Nagelkerke’s R^2^) of 0.02. The *OR* of the model indicates that students are 3% less likely to be cyberbullies (compared to those that are not involved) with every one-unit increase in their score on the openness trait. It was not possible to create an explanatory model for being a bully based on the aggression scores.

### 3.4. Prediction of Being a Victimized Cyberbully

It was possible to create two predictive models for being a victimized cyberbully, through personality and aggression (see Table 4), with this permitting the estimation of 76.9% of the cases (*χ*^2^ = 8.73; *p* = 0.00) and of 92.1% of the cases (*χ*^2^ = 9.85; *p* = 0.00), with a goodness of fit (Nagelkerke’s R^2^) of 0.03 and 0.06, respectively. The *OR* of the models indicates that students are 3% more likely to be victimized bullies (as compared to not involved individuals) with every one-unit increase in their score on the cordiality trait, and are 11% less likely to be victimized bullies with every one-unit increase in their score on anger.

## 4. Discussion

The objective of this study was to analyze the relationship between personality traits and aggression with cyberbullying behaviors in victims, bullies, victimized bullies and not-involved individuals. Based on the evidence found, in the case of victims, it was possible to partially confirm the first and second hypothesis. On the one hand, victims are characterized by extroverted, agreeable and open to new experiences profiles with low levels of neuroticism, while at the same time, they tend not to be very verbally aggressive. However, a logistic regression analysis confirmed that high scores on extraversion and low scores on verbal aggression explained their participation in this role, so sociable and verbally less aggressive preadolescents are more likely to be victims of cyberbullying. Although these findings are in agreement with past studies that have characterized victims by their high levels of extraversion, agreeableness and openness to experience [11,12], they refute other publications that have highlighted higher scores on neuroticism [40,41], as well as others that have found high levels of anger in cyberbullying victims [24,25]. The finding that the preadolescent cyberbullying victims had an adjusted and social profile, as well as the finding that extraversion explains their participation, may be related to the idea that more sociable subjects have a greater interest in relating with their peers through the Internet and social networks, and therefore, they may be more vulnerable to suffering from cyberbullying episodes, given their increased exposure to online situations [42]. As for the low level of verbal aggression as an explanatory and consequential factor, this may be due to the finding that victim harassment may be caused by a search for peer approval, and therefore, the fact that victims are less aggressive makes them easy targets for the cyberbullying behavior, since this guarantees a great benefit for the bullies: anonymity [43].

As for the bullies, it was possible to partially confirm the third and fourth hypothesis, since the bullies have been characterized by their lower scores on extraversion, agreeableness, conscientiousness and openness to experience, and higher scores on neuroticism, with openness to experience being an explanatory trait for engaging in cyberbullying when compared to not-involved individuals. These results are in line with those from past studies that found that bullies have unadjusted personalities with a low level of agreeableness, openness and high rates of neuroticism [4,11,12,13]. However, higher scores on aggression were not found, as was the case in past studies [21]. This may be due to the fact that, unlike traditional bullying, in the initial phases of cyberbullying, the bullies are more adjusted and have less aggressive profiles in terms of peer relations, perhaps due to the specific characteristics of the Internet. So, Chamarro, Beltrán, Oberst and Torres [44] affirmed that youths tend to feel invulnerable to the dangers of the Internet and are motivated by the immediate gratification and curiosity for stimulating and risky experiences, leading to impulse control difficulties. Furthermore, even those with well-adjusted personalities may be more vulnerable to risky behaviors [45]. Furthermore, since this is a primary school sample and thus, the cyberbullying cases are not chronic, it is possible that the internal motivations are not led by a sense of revenge, as was the case in the secondary school students [46], but rather, they may be caused by the lack of consequences or desire for group approval. So, the profiles are less aggressive. In any case, openness to experience has been found to be a protective factor against becoming a cyberbully, and therefore, students with more cultural interests and creativity have lower risk of becoming cyberbullies [11].

In the case of victimized bullies, as compared to “pure” bullies, they reveal significantly higher scores on agreeableness and openness to experience. However, they have similar scores as the “pure” victims on the other personality traits and on the distinct manifestations of aggression. So, victimized bullies have a personality profile that is similar to that of the victims, while agreeableness and low anger levels are the factors explaining being a victimized bully. So, despite the fact that past studies have suggested that adolescent victimized bullies have the lowest levels of social skills [47], our results suggest that during preadolescence, when cyberbullying has yet to become chronic, minors with adjusted personality characteristics, such as sensitivity towards others and lower anger levels, have a greater risk of developing a victimized bully profile. This contradicts studies carried out on the adolescent population which concluded that victimization may affect student’s empathy levels and participation in equally aggressive behaviors [48], and victimized bullies have higher aggression levels [18,21].

As for the not involved individuals, novel information has been provided with regard to personality traits. However, the fifth hypothesis, on the frequency of aggressive behaviors, was rejected. So, children that are not involved in cyberbullying, when compared to the victimized bullies, present lower scores on extraversion, agreeableness, conscientiousness and openness to experience, and higher scores on neuroticism. In addition, they have an aggressive profile of verbal behavior and anger, higher than that of the victimized bully but similar to that of the “pure” bully and victim. These findings suggest that bystanders or not-involved individuals, may have certain personality characteristics that are similar to those of the victims and bullies, in terms of aggressive behaviors. This phenomenon may be explained based on their positioning either in support of or against the bully. Schultze-Krumbholz, Hess, Pfetsch and Scheithauer found that “aggressive defenders” were more likely to develop reactive aggression, whereas “prosocial defenders” were less likely. The results, therefore, conclude that within the role of the not involved individual, distinct profiles appear to exist, based on the aggressive cyberbullying behaviors [18]. In addition, González suggested that many children believe that victims deserve this treatment [49], therefore, it is possible that cyberbullying has become a regular aspect of their daily lives, causing no feelings of remorse, with bystanders, thereby, becoming accomplices of the bullies [19]. This finding agrees with reflections made by other authors who have found that youth often consider insults and threats made over the Internet to be a regular part of peer communication and interaction [50], leading to the potential normalization of cyberbullying behaviors, especially between minors who are not directly involved in the cyberbullying cases.

This study has certain limitations. First, the results cannot be generalized to Spanish students at other educational levels (Secondary Education and Higher Education), given the selected sample. Therefore, future studies should analyze the results for other educational levels. In addition, the cross-sectional design used in the study makes it impossible to establish causal relationships, so it is recommended that experimental studies and longitudinal designs be employed to provide information on the evolution of the phenomenon over time. Moreover, considering the moderate relationship between bullying and cyberbullying, future studies should consider the joint relationship between both constructs in Primary Education students. Finally, given the ongoing innovation taking place in the technological fields, (e.g., applications, social networks, devices), future studies should include potential new ways of perpetrating this harassment, that have yet to be considered.

## 5. Conclusions

This study provides novel information that is of great relevance to the study of cyberbullying in Primary Education, not only given the limited evidence available for this age range, but also thanks to the results on the distinct cyberbullying roles (bullies, victims, victimized bullies and not-involved individuals) with regard to personality and aggression. It should be noted that the personality characteristics of preadolescents may vary once reaching adolescence [51]; therefore, it is necessary to continue to study this in order to corroborate whether this personality structure is a result of the pre-adolescence stage or is related to participation in these events. In any case, the results support the idea that, between the ages of 10 and 13, students who are less aggressive and have an adaptive personality structure are also at risk of suffering from, engaging in and observing cyberbullying. These findings could be considered in the elaboration of cyberbullying prevention programs that are so necessary in the school context [52,53].

## Figures and Tables

**Table 1:** Differences in means and standard deviations for personality traits and aggression between students who are pure victims and bullies, victimized bullies and not involved in cyberbullying.

	Cybervictim	Cyberbully	Cyberbully-Victim	Not Involved	Statistical Significance	
	(*n* = 100)	(*n* = 38)	(*n* = 103)	(*n* = 307)		
	*M (D.T.)*	*M (D.T.)*	*M (D.T.)*	*M (D.T.)*	*F*	*p*
Personality						
Extraversion	42.36 (8.33)	37.80 (12.03)	41.95 (9.87)	38.70 (10.14)	4.87	0
Agreeableness	42.19 (11.77)	36.32 (14.89)	43.98 (14.35)	38.80 (13.10)	5.04	0
Conscientiousness	40.92 (10.91)	36.32 (15.47)	42.60 (12.82)	38.68 (11.80)	3.52	0.01
Neuroticism	37.81 (10.05)	40.87 (13.04)	36.03 (10.85)	40.30 (11.28)	4.04	0
Openness	41.71 (8.64)	35.87 (14.31)	42.37 (10.87)	38.07 (10.86)	6.23	0
Aggression						
Hostility	12.62 (7.23)	13.27 (6.89)	11.43 (8.14)	14.50 (6.49)	2.64	0.05
Verbal aggression	4.45 (3.19)	4.94 (4.53)	4.46 (4.22)	6.50 (4.27)	4.94	0
Anger	9.65 (4.23)	9.16 (5.33)	8.30 (5.73)	11.78 (5.24)	6.44	0
Physical aggression	8.52 (6.38)	9.27 (8.65)	7.76 (7.17)	10.69 (6.89)	2.61	0.05

**Table 2:** Results from the binary logistic regression for the probability of being a cyberbullying victim.

Model	Predictor Variable	B	S.E.	Wald	*p*	*OR*	C.I. 95%
Personality	Extraversion	0.02	0.01	5.81	0.01	1.03	1.01–1.05
	Constant	−2.44	0.51	22.57	0.00	0.08	
Aggression	Verbal Aggression	−0.11	0.05	5.92	0.01	0.89	0.82–0.98
	Constant	−1.54	0.27	32.40	0.00	0.21	

B = coefficient; S.E. = standard error; p = probability; OR = odds ratio; C.I. = confidence interval at 95%.

**Table 3:** Results from the binary logistic regression for the probability of being a cyberbully.

Model	Predictor Variable	B	S.E.	Wald	*p*	*OR*	C.I. 95%
Personality	Openness	−0.03	0.02	4.08	0.04	0.97	0.93–0.99
	Constant	−1.27	0.65	3.87	0.04	0.28	

B = coefficient; S.E. = standard error; p = probability; OR = odds ratio; C.I. = confidence interval at 95%.

**Table 4:** Results from the binary logistic regression for the probability of being a victimized cyberbully.

Model	Predictor Variable	B	S.E.	Wald	*p*	*OR*	C.I. 95%
Personality	Cordiality	0.03	0.01	8.36	0.00	1.03	1.01–1.04
	Constant	−2.27	0.39	32.69	0.00	0.10	
Aggression	Anger	−0.12	0.04	9.15	0.00	0.89	0.82–0.96
	Constant	−1.30	0.38	11.46	0.00	0.27	

B = coefficient; S.E. = standard error; p = probability; OR = odds ratio; C.I. = confidence interval at 95%.
